# Two birds with one stone: human SIRPα nanobodies for functional modulation and *in vivo* imaging of myeloid cells

**DOI:** 10.3389/fimmu.2023.1264179

**Published:** 2023-12-18

**Authors:** Teresa R. Wagner, Simone Blaess, Inga B. Leske, Desiree I. Frecot, Marius Gramlich, Bjoern Traenkle, Philipp D. Kaiser, Dominik Seyfried, Sandra Maier, Amélie Rezza, Fabiane Sônego, Kader Thiam, Stefania Pezzana, Anne Zeck, Cécile Gouttefangeas, Armin M. Scholz, Stefan Nueske, Andreas Maurer, Manfred Kneilling, Bernd J. Pichler, Dominik Sonanini, Ulrich Rothbauer

**Affiliations:** ^1^ NMI Natural and Medical Sciences Institute at the University of Tübingen, Reutlingen, Germany; ^2^ Pharmaceutical Biotechnology, Eberhard Karls University Tübingen, Tübingen, Germany; ^3^ Werner Siemens Imaging Center, Department of Preclinical Imaging and Radiopharmacy, University of Tübingen, Tübingen, Germany; ^4^ German Cancer Consortium (DKTK) and German Cancer Research Center (DKFZ) partner site Tübingen, Tübingen, Germany; ^5^ Preclinical Models & Services, genOway, Lyon, France; ^6^ Department of Immunology, Institute of Cell Biology, University of Tübingen, Tübingen, Germany; ^7^ Cluster of Excellence iFIT (EXC2180) “Image-Guided and Functionally Instructed Tumor Therapies”, University of Tübingen, Tübingen, Germany; ^8^ Livestock Center of the Faculty of Veterinary Medicine, Ludwig Maximilians University Munich, Oberschleissheim, Germany; ^9^ Department of Dermatology, University of Tübingen, Tübingen, Germany; ^10^ Department of Medical Oncology and Pneumology, University of Tübingen, Tübingen, Germany

**Keywords:** nanobodies (Nbs), SIRPalpha, myeloid cells, PET imaging tracer, immune checkpoint inhibitor (ICI), theranostics

## Abstract

Signal-regulatory protein α (SIRPα) expressed by myeloid cells is of particular interest for therapeutic strategies targeting the interaction between SIRPα and the “don’t eat me” ligand CD47 and as a marker to monitor macrophage infiltration into tumor lesions. To address both approaches, we developed a set of novel human SIRPα (hSIRPα)–specific nanobodies (Nbs). We identified high-affinity Nbs targeting the hSIRPα/hCD47 interface, thereby enhancing antibody-dependent cellular phagocytosis. For non-invasive *in vivo* imaging, we chose S36 Nb as a non-modulating binder. By quantitative positron emission tomography in novel hSIRPα/hCD47 knock-in mice, we demonstrated the applicability of ^64^Cu-hSIRPα-S36 Nb to visualize tumor infiltration of myeloid cells. We envision that the hSIRPα-Nbs presented in this study have potential as versatile theranostic probes, including novel myeloid-specific checkpoint inhibitors for combinatorial treatment approaches and for *in vivo* stratification and monitoring of individual responses during cancer immunotherapies.

## Introduction

During tumor development, there is a continuous exchange between malignant cells, neighboring parenchymal cells, stromal cells, and immune cells. Together with the extracellular matrix and soluble mediators, these cells constitute the tumor microenvironment (TME). The composition of the immune infiltrate within the TME largely determines cancer progression and sensitivity to immunotherapies (1). Myeloid cells are known to regulate T-cell responses, thereby bridging innate and adaptive immunity (2–4). Tumor cells further utilize myeloid cells to create a pro-tumorigenic milieu by exploiting their ability to produce immune-regulating mediators (e.g., interleukin-6 and tumor necrosis factor), growth factors influencing tumor proliferation and vascularization (e.g., transforming growth factor–β and vascular endothelial growth factor), as well as matrix-degrading enzymes (e.g., matrix metalloproteinases) (5). Within the myeloid cell population, tumor-associated macrophages (TAMs) are highly abundant, and widely varying densities of up to 50% of the tumor mass are observed (6). At the same time, depending on their polarization state, TAMs exhibit partially opposing effects either as key drivers for tumor progression or by exerting potent antitumor activity (7, 8). Consequently, monitoring tumor infiltration of TAMs is of great importance for patient stratification and companion diagnostic (9–11), and targeted recruitment or activation of anti-tumor TAMs opens new strategies to achieve persisting anti-tumor immune responses (12).

In this context, the myeloid-specific immune checkpoint signal-regulatory protein α (SIRPα), expressed by monocytes, macrophages, dendritic cells, and neutrophils (13, 14), represents an interesting theranostic target. Interaction with its ligand CD47, a “marker of self” virtually expressed by all cells of the body, mediates a “don’t eat me” signal that inhibits phagocytosis, and prevents subsequent autoimmune responses. Exploiting this physiological checkpoint, tumor cells often upregulate CD47 and thereby escape recognition and removal by macrophages (15, 16). Similarly, enhanced expression of SIRPα by intratumoral monocytes/macrophages has recently been shown to be associated with poorer survival in follicular lymphoma, colorectal cancer, intrahepatic cholangiocarcinoma, and esophageal cancer (17–19).

To address the potential as a biomarker and immune modulating target, we generated human SIRPα (hSIRPα)–specific nanobodies (Nbs) for diagnostic and potential therapeutic applications. Nbs are single-domain antibodies derived from heavy-chain antibodies of camelids (20, 21) and have emerged as versatile biologicals for therapeutic as well as diagnostic purposes (22–24). Compared with conventional antibodies, Nbs exhibit superior characteristics concerning chemical stability, solubility, and tissue penetration due to their small size and compact folding (20). Following a binary screening strategy, in-depth biochemical characterization, epitope mapping, and functional studies, we identified two hSIRPα-Nb subsets that either block the hSIRPα/hCD47 interface or serve as inert probes for molecular imaging.

## Results

### Selection of high-affinity anti-human SIRPα nanobodies

**Figure 1:**
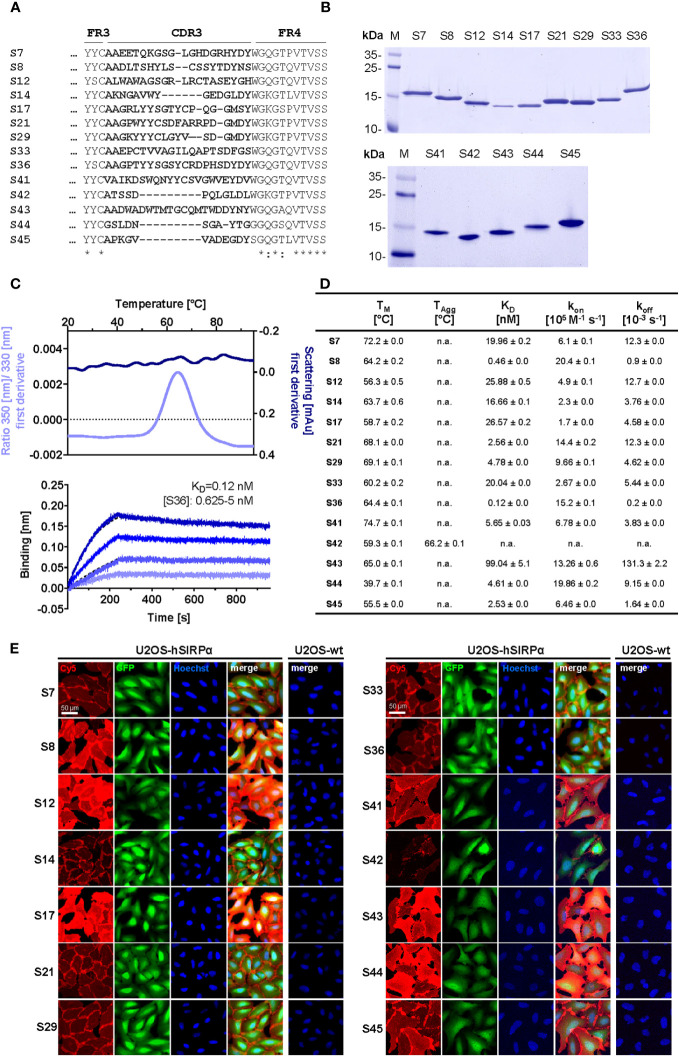
Biochemical characterization of hSIRPα Nbs. **(A)** Amino acid (aa) sequences of the complementarity-determining region (CDR) 3 from 14 unique hSIRPα Nbs (complete sequences shown in **Supplementary Table 1**) identified by a bidirectional screening strategy. Nbs S7 to S36 were selected against full-length hSIRPα and Nbs S41 to 45 against domain 1 of hSIRPα (hSIRPαD1). **(B)** Recombinant expression and purification of hSIRPα Nbs using immobilized metal affinity chromatography (IMAC) and size exclusion chromatography (SEC). Coomassie staining of purified Nbs is shown. **(C)** Stability analysis using nano–differential scanning fluorimetry (nanoDSF) displaying fluorescence ratio (350 nm/330 nm) and light intensity loss due to scattering shown as first derivative exemplarily shown for Nb S36 (upper panel). Data are shown as mean value of three technical replicates. BLI-based affinity measurements exemplarily shown for Nb S36 (bottom panel). Biotinylated hSIRPα was immobilized on streptavidin biosensors. Kinetic measurements were performed using four concentrations of purified Nbs ranging from 0.625 nM to 5 nM (displayed with gradually darker shades of color). The binding affinity (K_D_) was calculated from global 1:1 fits shown as dashed lines. **(D)** Summary table of stability and affinity analysis of selected hSIRPα Nbs. Melting temperature (T_M_) and aggregation temperature (T_Agg_) determined by nanoDSF shown as mean ± SD of three technical replicates. Affinities (K_D_), association constants (*k*_on_), and dissociation constants (*k*_off_) determined by BLI using four concentrations of purified Nbs shown as mean ± SD. **(E)** Representative images of hSIRPα and GFP-coexpressing U2OS cells stained with hSIRPα Nbs of three technical replicates. Images show individual Nb staining detected with anti-VHH-Cy5 (red), intracellular IRES-derived GFP signal (green), nuclei staining (Hoechst, blue), and merged signals; scale bar, 50 µm.

To generate Nbs against hSIRPα that can be used either as probes for diagnostic imaging or to modulate interaction with human CD47, we immunized an alpaca (*Vicugna pacos*) with the recombinant extracellular portion of hSIRPα and established an Nb phagemid library (2 × 10^7^ clones). This Nb library was subjected to phage display–based selection campaigns targeting either the entire extracellular portion or exclusively domain 1 (D1) of hSIRPα (hSIRPαD1) to guide the selection of Nbs that specifically block the hSIRPα/hCD47 interaction. Sequencing of individual clones resulted in 14 unique hSIRPα Nbs with high diversity in the complementarity-determining region 3 (CDR3) (**Figure 1A**; **Supplementary Table 1**). Nbs S7 to S36 were derived from the full-length hSIRPα screening, whereas Nbs S41 to S45 were identified as hSIRPαD1 binders. Individual Nbs were produced in *Escherichia coli* (*E. coli*) and isolated with high purity (**Figure 1B**). Folding stability of all Nbs was analyzed by differential scanning fluorimetry. For 12 out of the 14 Nb candidates, melting temperatures ranging from ~55°C to ~75°C without aggregation (**Figures 1C, D**; **Supplementary Figure 1A**) were determined, whereas affinity measurements against recombinant hSIRPα by biolayer interferometry (BLI) revealed K_D_ values between ~0.12 nM and ~27 nM for 11 out of the 12 Nbs (**Figures 1C, D**; **Supplementary Figure 1B**). In addition, live-cell immunofluorescence staining of U2OS - Human Bone Osteosarcoma Epithelial Cells stably expressing full-length hSIRPα showed that all selected Nbs recognize hSIRPα localized at the plasma membrane (**Figure 1E**; **Supplementary Figure 2A**).

### Domain mapping of hSIRPα Nbs

**Figure 2:**
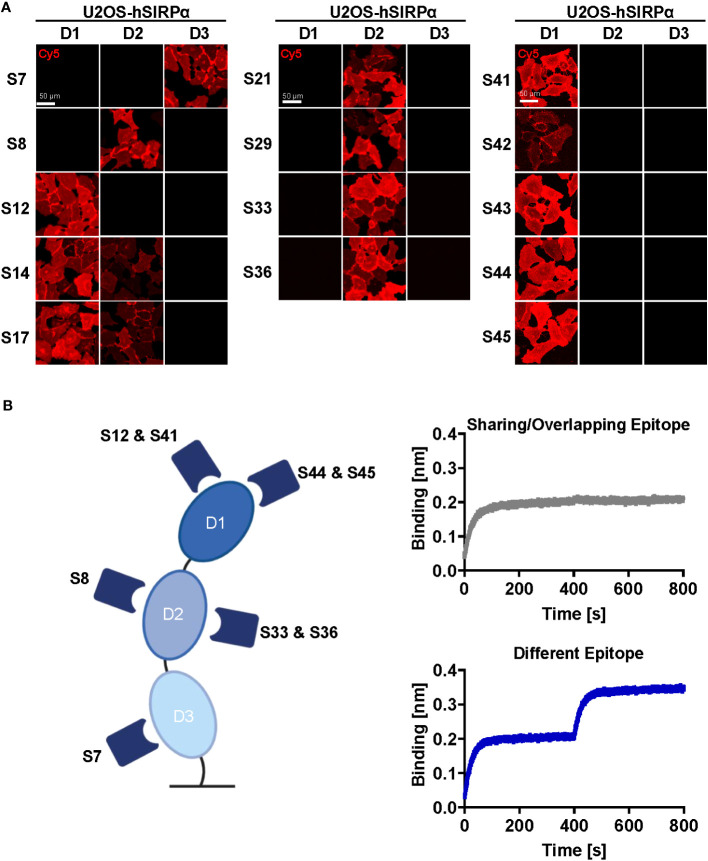
Epitope characterization of hSIRPα Nbs. **(A)** Domain mapping analysis by immunofluorescence staining with hSIRPα Nbs on U2OS cells displaying human hSIRPα domain 1 (D1), domain 2 (D2), or domain 3 (D3) at their surface. Representative images of live cells stained with individual Nbs in combination with Cy5-labeled anti-VHH of three technical replicates are shown; scale bar, 50 µm. **(B)** Epitope binning analysis of hSIRPα Nbs by BLI. Graphical summary of epitope binning analysis on the different hSIRPα domains (left panel). Representative sensograms (n = 1) of combinatorial Nb binding to recombinant hSIRPα on sharing/overlapping epitopes or on different epitopes (right panel).

Whereas Nbs targeting hSIRPαD1 have a higher chance to block interaction with CD47, Nbs targeting domain D2 or D3 (hSIRPαD2 and hSIRPαD3) might be functionally inert, which is preferable for diagnostic approaches. Thus, we assessed domain specificity using U2OS cells expressing the individual domains of hSIRPα by immunofluorescence staining (**Figure 2A**, **Supplementary Figure 2B**). Eight Nbs (S12, S14, S17, S41, S42, S43, S44, and S45) stained hSIRPαD1, whereas Nbs S14 and S17 additionally stained hSIRPαD2. Five Nbs (S8, S21, S29, S33, and S36) revealed specific binding to hSIRPαD2, whereas only Nb S7 stained cells expressing hSIRPαD3. On the basis of their respective production yield, stability, affinity, domain specificity, and developability, we selected Nbs S7, S8, S12, S33, S36, S41, S44, and S45 for further characterization. To determine the diversity of epitopes recognized by this subset in more detail, we performed an epitope binning analysis using BLI (**Figure 2B**; **Supplementary Figures 3A, B**). On the basis of the results, we grouped the Nbs according to shared or overlapping epitopes and found two groups each for hSIRPαD1-targeting (Nbs S12 and S41 and Nbs S44 and S45) and hSIRPαD2-targeting (Nb S8 and Nbs S33 and S36) Nbs (**Supplementary Figures 3A, B**).

### Specificity of hSIRPα Nbs for allelic variants and closely related SIRP family members

**Figure 3:**
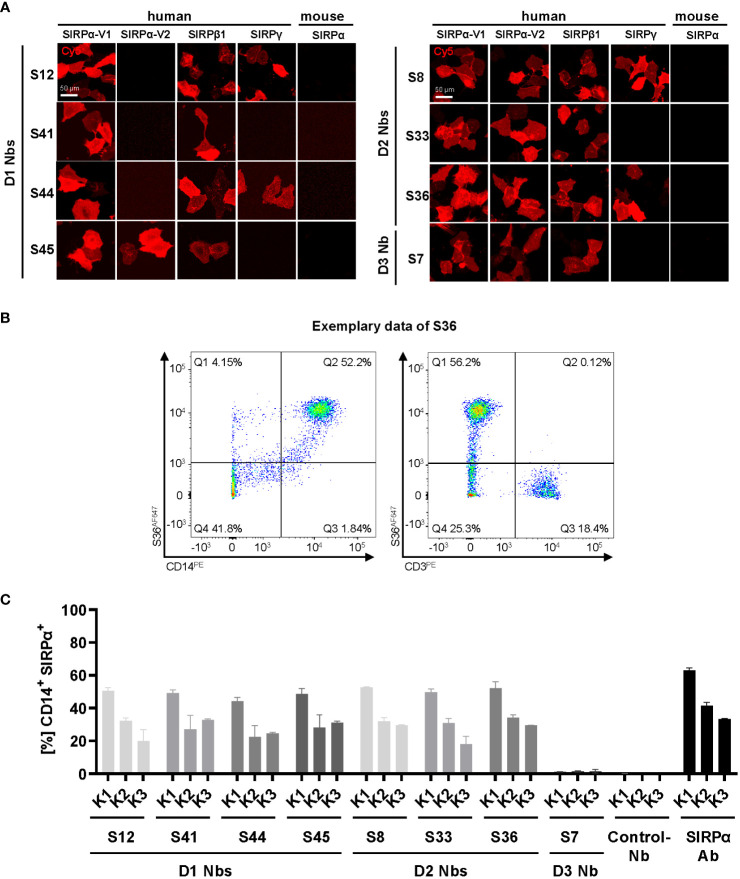
Cross-reactivity and binding specificity of hSIRPα Nbs. **(A)** Cross-reactivity analysis of hSIRPα Nbs by immunofluorescence staining on U2OS cells displaying hSIRPα-V1, hSIRPα-V2, hSIRPβ1, hSIRPγ, or mouse SIRPα at their surface. Representative images of live cells stained with individual Nbs in combination with Cy5-labeled anti-VHH are shown of three technical replicates; scale bar, 50 µm. **(B)** Flow cytometry analysis of human peripheral blood mononuclear cells (PBMCs) stained with fluorescently labeled hSIRPα Nbs (AlexaFluor 647, AF647). Flow cytometry plots of Nb S36 staining on CD14^+^ and CD3^+^ PBMC populations derived from human donor K1 are shown as an example. **(C)** Flow cytometry analysis of hSIRPα Nbs staining CD14^+^ PBMCs of three different human donors (K1, K2, and K3). As control, PBMCs were stained with a Pep Nb (Control-Nb) and a SIRPα-antibody (positive control). Data are presented as mean ± SD of three technical replicates.

hSIRPα belongs to the hSIRP family of immune receptors, which also includes the highly homologous activating receptor hSIRPβ1 present on macrophages, and the decoy receptor hSIRPγ, which is expressed mainly on T cells (14). Moreover, hSIRPα allelic variants, hSIRPαV1 and hSIRPαV2, are expressed either homozygously (v1/v1 or v2/v2) or heterozygously (v1/v2) (25). To address potential cross-reactivity, binding of selected hSIRPα Nbs to hSIRPβ1, hSIRPγ, the hSIRPα variants hSIRPα-V1 and hSIRPα-V2, and murine SIRPα was visualized using immunofluorescence staining (**Figure 3A**; **Supplementary Figure 2C**). Cellular imaging revealed that all Nbs recognized the homologous hSIRPβ1, whereas hSIRPγ was detected with Nbs S12 and S44 (both hSIRPαD1-targeting Nbs) as well as Nbs S8 and S36 (both hSIRPαD2-targeting Nbs). Furthermore, all hSIRPαD2- and D3-targeting Nbs recognized hSIRPα-V1 and hSIRPα-V2, whereas S45 was the only hSIRPαD1-targeting Nb to show binding to both variants. Notably, none of the selected Nbs revealed any cross-reactivity towards murine SIRPα.

### Binding of hSIRPα Nbs to primary human monocyte/macrophage cells

To evaluate whether our hSIRPα Nbs recognize endogenously expressed hSIRPα, we performed flow cytometry analysis of peripheral blood mononuclear cells (PBMCs) from three different donors (K1–K3). In addition to the monocyte/macrophage marker CD14, we also included the T-cell marker CD3 to evaluate potential recognition of T cells by hSIRPγ–cross-reactive Nbs (**Figure 3B**). All hSIRPα Nbs, except S7, stained comparably on CD14^+^ PBMCs from all tested donors, whereas none of the Nbs stained CD3^+^ T cells (**Figures 3B, C**).

Considering our binary strategy to select hSIRPα Nbs (i) that are eligible to inhibit the hSIRPα/hCD47 interaction and (ii) as probes for positron emission tomography (PET)–based *in vivo* imaging of myeloid cells, we divided the identified Nbs into two subgroups. In the following, hSIRPαD1-targeting Nbs S12, S41, S44, and S45 were further investigated with respect to their inhibitory properties, and hSIRPαD2-targeting Nbs S8, S33, and S36 for their applicability as *in vivo* imaging probes.

### hSIRPαD1 Nbs functionally block the interaction with hCD47

**Figure 4:**
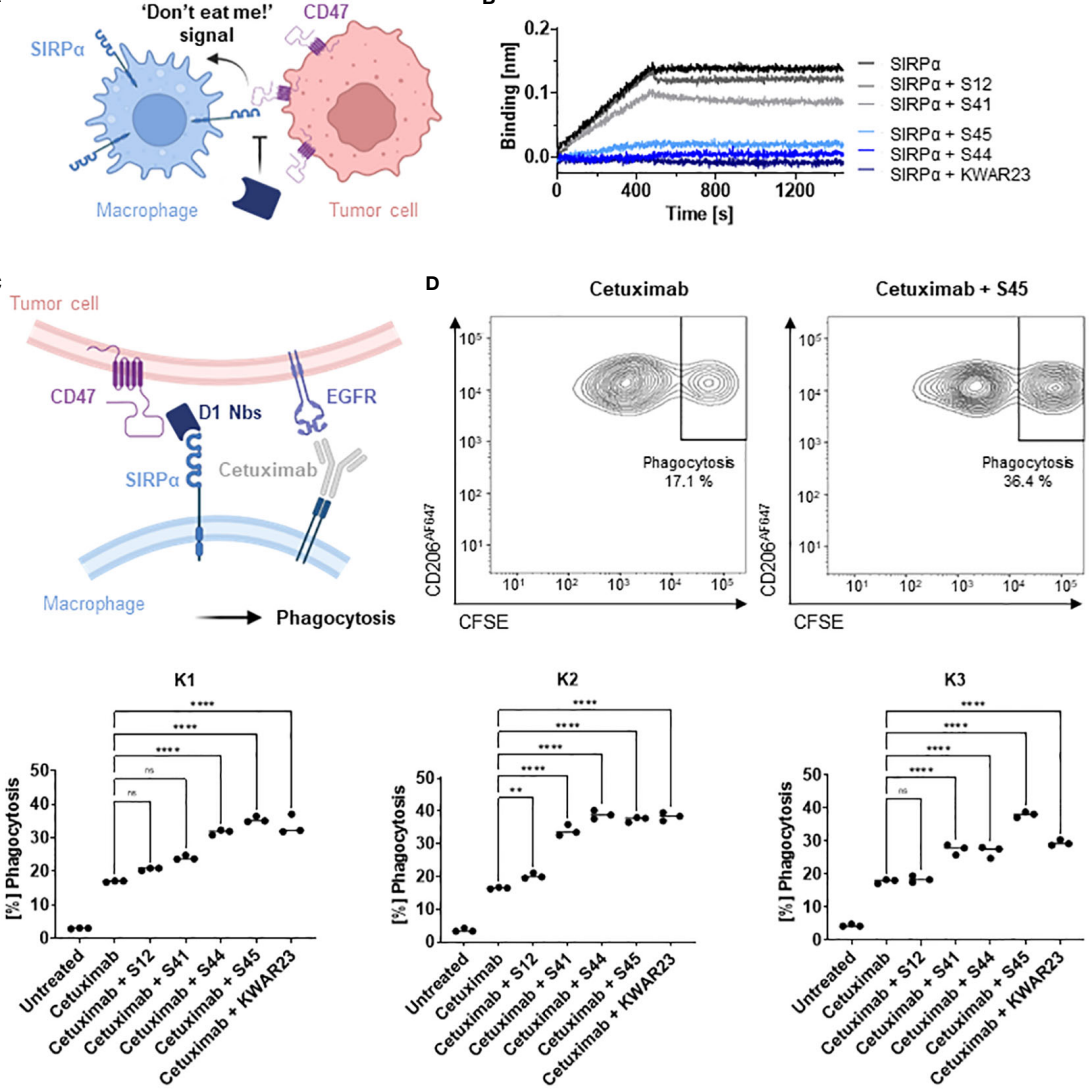
Potential of hSIRPαD1 Nbs to augment phagocytosis of tumor cells. **(A)** Graphical illustration of hSIRPα/hCD47 interaction leading to suppression of macrophage-mediated phagocytosis of tumor cells. **(B)** Competition analysis of hSIRPα-binding to hCD47 in the presence of hSIRPαD1 Nbs (S12, S41, S44, and S45) by BLI (n = 1). Biotinylated hCD47 was immobilized on streptavidin biosensors, and a mixture of 20 nM hSIRPα and 250 nM of hSIRPαD1 Nbs or 5 nM of KWAR23 was applied to elucidate potential inhibition of hSIRPα binding to hCD47. **(C)** Schematic illustration of macrophage-mediated phagocytosis of tumor cells by hSIRPαD1 Nbs and tumor-opsonizing antibodies (e.g., the anti-EGFR antibody cetuximab). **(D)** Phagocytosis of CFSE–labeled DLD-1 cells by human monocyte-derived macrophages. A representative flow cytometry plot of the phagocytosis assay of cetuximab only and combinatorial treatment of cetuximab and hSIRPα Nb S45 with donor K1–derived macrophages is shown. **(E)** Quantitative analysis of the phagocytosis assay. Percent of phagocytosis of CFSE-labeled DLD-1 cells analyzed for macrophages derived from three different donors (K1, left; K2, center; K3, right) in different conditions is shown. Data are shown as individual and mean value of three technical replicates. p < 0.05 was considered statistically significant (*) and marked as ** for p < 0.01, *** for p < 0.001, and **** for p < 0.0001; non-significant results were marked with ns.

To evaluate potential inhibition of the interaction between hSIRPα and hCD47 (**Figure 4A**), we first performed a competitive BLI-based binding assay. As control, we used the anti–hSIRPα-blocking antibody KWAR23 (26). After incubation with Nb S44 or S45, binding of hSIRPα to CD47 was inhibited to a similar extent as upon addition of KWAR23; whereas only partial blocking was observed for S41, S12 showed no effect (**Figure 4B**). For functional analysis, we next tested the ability of hSIRPαD1-targeting Nbs to potentiate macrophage-mediated antibody-dependent cellular phagocytosis (ADCP) (**Figure 4C**). To this end, human monocyte-derived macrophages (MDMs) isolated from three different donors (K1–K3) were incubated with Epidermal Growth Factor Receptor (EGFR^+^) expressing human colorectal adenocarcinoma DLD-1 cells preloaded with carboxyfluorescein diacetate succinimidyl ester (CFSE) alone or in the presence of the opsonizing EGFR-specific antibody cetuximab and hSIRPαD1-targeting Nbs or the KWAR23 antibody as positive control. The degree of ADCP was determined on the basis of the detection of CD206^+^CFSE^+^ cells by flow cytometry analysis (**Figure 4D**). For all tested donors, macrophages exhibited minimal phagocytosis of DLD-1 cells without treatment, whereas phagocytic activity was significantly increased upon addition of cetuximab. In the presence of the hSIRPα-blocking antibody KWAR23, phagocytosis was further induced, which is in line with previous findings (26). Similarly, the hSIRPα-blocking Nbs S44 and S45 augmented ADCP in all three tested donors, whereas Nb S12 and S41 only revealed limited effect on macrophage-mediated phagocytosis (**Figure 4E**). From these results, we concluded that Nbs S44 and S45 represent promising candidates for further development as novel hSIRPα/CD47-inhibitory biologicals for potential therapeutic applications.

### Inert hSIRPα-S36 Nb as lead candidate for non-invasive *in vivo* imaging

For the application as non-invasive PET tracer, immunologically inert hSIRPα Nbs are preferred. Thus, we selected Nbs S8, S33, and S36, which bind to hSIRPαD2, and performed a detailed analysis of the recognized epitopes by hydrogen-deuterium exchange mass spectrometry (HDX-MS). All selected Nbs recognized three-dimensional epitopes within hSIRPαD2, which are spatially distant from the hSIRPα/hCD47 interface (**Supplementary Table 2**; **Supplementary Figures 4A, B**). In particular, S36 Nb showed the strongest deuteration protection (<−15%) for amino acid (aa) D163 to L187 and aa H202 to G207 of hSIRPα, whereas an additional slightly lower protection was observed for the region ranging from aa C140 to K153 (**Supplementary Figures 4A, B**). Considering its detailed epitope mapping, strong binding affinity, and good production yield, we selected S36 Nb as the lead candidate for imaging.

For radiolabeling, we conceived a novel protein engineering approach that enables site-specific chemical conjugation. We first adapted the sequence of the original S36 Nb by replacing all four lysine residues with arginine (hSIRPα-S36_K>R_ Nb) (**Supplementary Figure 5A**) and conjugated the chelator via isothiocyanate (p-NCS-benzyl-NODA-GA) to the remaining primary NH_2_-group at the N-terminus (**Supplementary Figure 5A**). The hSIRPα-S36_K>R_ Nb was producible with comparable yield and purity to the original version in *E.coli* (**Supplementary Figure 5B**) and efficient site-specific chelator conjugation (~96%) was confirmed by mass spectrometry. Most importantly, the hSIRPα-S36_K>R_ Nb showed comparable affinities and characteristics to the original S36 Nb (**Supplementary Figures 5C–E**). Finally, we examined the hSIRPα-S36_K>R_ Nb in the macrophage-dependent phagocytosis assay. Here, we observed a minor induction of macrophage-dependent phagocytosis that is comparable to the effect of the non-blocking Nb S12 (**Supplementary Figure 5F**; **Figure 4E**). From these results, we concluded that hSIRPα-S36_K>R_ Nb, represents a lead candidate suitable for non-invasive *in vivo* PET imaging of SIRPα expression.

### PET/MR imaging with ^64^Cu-hSIRPα-S36_K>R_ Nb

**Figure 5:**
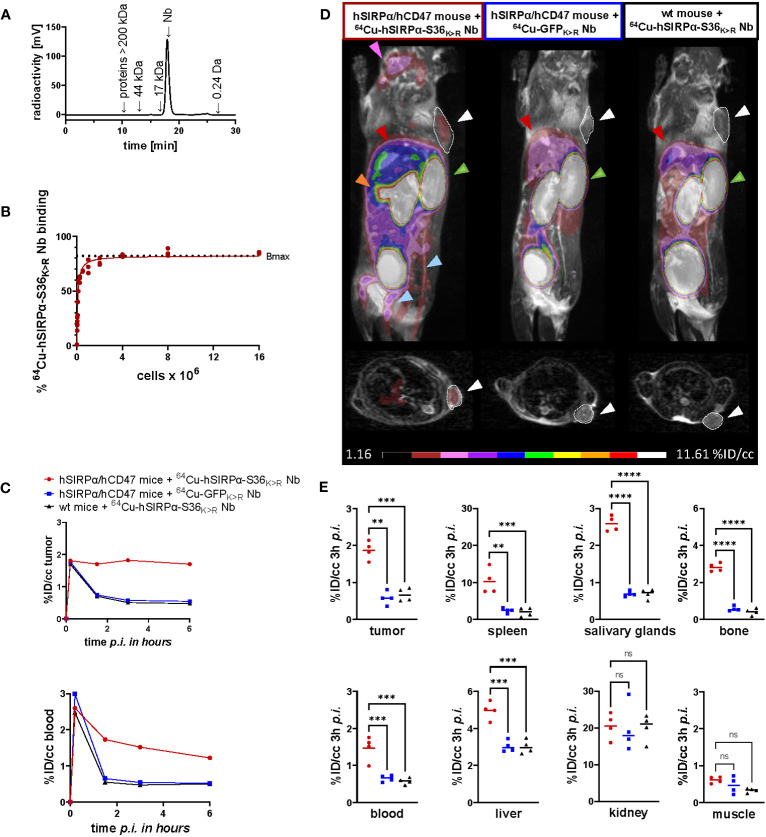
PET imaging with ^64^Cu-hSIRPα-S36_K>R_ Nb. **(A)** Radiochemical purity of ^64^Cu-hSIRPα-S36_K>R_ Nb was assessed using high-performance liquid chromatography (HPLC). **(B)** Antigen excess binding assay to determine the maximum binding (Bmax) of ^64^Cu-hSIRPα-S36_K>R_ Nb, referred to as immunoreactive fraction. ^64^Cu-hSIRPα-S36_K>R_ Nb (1 ng) was applied to an increasing number of HT1080-hSIRPα cells of three technical replicates and binding curves were analyzed using the one-site nonlinear regression model. **(C)** Quantification of ^64^Cu-hSIRPα-S36_K>R_ Nb tumor and blood uptake of *s.c.* MC38-hCD47 colon carcinoma-bearing hSIRPα/hCD47 mice over 6 h after injection. ^64^Cu-hSIRPα-S36_K>R_ Nb accumulation is compared to the control groups injected with control Nb or in MC38 wt mice injected with ^64^Cu-hSIRPα-S36_K>R_ Nb. The resulting values were decay-corrected and presented as percentage of injected dose per cubic centimeter (%ID/cc). Representative data of one animal per group is shown. **(D)** Representative fused MIP (maximum intensity projection) PET/MR images of mice 3 h after ^64^Cu-hSIRPα-S36_K>R_ (n = 4) or control Nb injection (each n = 4). PET signal in hSIRPα expressing myeloid cell–rich organs is compared to both control groups. Sites with increased ^64^Cu-hSIRPα-S36_K>R_ Nb uptake are marked by colored arrows indicating the tumor (white and outlined), spleen (orange), bone (blue), salivary glands (purple), kidneys (green), and liver (red). In addition, axial sections of PET/MR images are shown where the tumors are highlighted with white circles and arrows. **(E)** Quantification of ^64^Cu-hSIRPα-S36_K>R_ Nb in hSIRPα expressing myeloid cell–rich organs. High accumulation was also detected in sites of excretion, namely, the kidney and liver. The resulting values were decay-corrected and presented as percentage of injected dose per cubic centimeter (%ID/cc). Data are shown as individual plots and mean value (n = 4). p < 0.05 was considered statistically significant (*) and marked as ** for p < 0.01, *** for p < 0.001, and **** for p < 0.0001; non-significant results were marked with ns.

For *in vivo* validation, the hSIRPα-S36_K>R_ Nb and the non-specific GFP_K>R_ Nb (6) as control were radiolabeled with ^64^Cu yielding high radiolabeling efficiencies of ≥95% (**Figure 5A**) and an *in vitro* immunoreactive fraction of ~82% (B_max_) of the ^64^Cu-labeled hSIRPα-S36_K>R_ Nb (^64^Cu-hSIRPα-S36_K>R_ Nb) to HT1080 hSIRPα knock-in (KI) (HT1080-hSIRPα) cells (**Figure 5B**).

To visualize the distribution of hSIRPα-positive cells in a tumor-relevant system, we employed a novel immunocompetent hSIRPα/hCD47 KI mouse model (hSIRPα/hCD47 mice), expressing the extracellular domain of hSIRPα, and C57BL/6 wild-type (wt) mice as controls. In both models, tumors were generated by subcutaneous (*s.c.*) injection of hCD47-overexpressing MC38 (MC38-hCD47) colon adenocarcinoma cells. Nine days after tumor inoculation, we intravenously (*i.v.*) injected ^64^Cu-hSIRPα-S36_K>R_ Nb into both groups. As additional control, the non-specific ^64^Cu-GFP_K>R_ Nb was injected in tumor-bearing hSIRPα/hCD47 mice. Non-invasive *in vivo* PET/MR imaging revealed a strongly enhanced ^64^Cu-hSIRPα-S36_K>R_ Nb accumulation in the tumors of hSIRPα/hCD47 mice within the first minutes after injection, which remained stable at a high level for 6 h. In contrast, both control groups, ^64^Cu-GFP_K>R_ Nb–injected hSIRPα/hCD47 mice and ^64^Cu-hSIRPα-S36_K>R_ Nb–injected wt mice, showed rapid tracer clearance in the tumors and blood (**Figure 5C**). Importantly, ^64^Cu-hSIRPα-S36_K>R_ Nb–injected hSIRPα/hCD47 mice exhibited a constantly higher PET signal in the blood over time, indicating a specific binding to circulating hSIRPα^+^ myeloid cells (**Figure 5C**). Quantification of the PET images 3 h after injection revealed a significantly higher uptake in the tumors of hSIRPα/hCD47 mice (1.89 ± 0.09%ID/cc) compared with that of wt mice (0.60 ± 0.05%ID/cc) and to ^64^Cu-GFP_K>R_ Nb–injected hSIRPα/hCD47 mice (0.57 ± 0.05%ID/cc) (**Figures 5C–E**). Furthermore, we observed a ~7-fold enhanced uptake in the spleen, a ~2-fold enhanced uptake in the blood and liver, and a ~3-fold enhanced uptake in the salivary glands and bone in hSIRPα/hCD47 mice (**Figures 5D, E**), whereas no significant differences were identified in the kidney and the muscle tissue between the ^64^Cu-hSIRPα-S36_K>R_ Nb–injected hSIRPα/hCD47 mice and both control groups (**Figures 5D, E**). From these results, we concluded that the novel ^64^Cu-hSIRPα-S36_K>R_ Nb–based PET tracer is applicable to visualize and monitor the distribution of SIRPα^+^ cells by non-invasive *in vivo* imaging.

## Discussion

Myeloid cells, particularly macrophages, frequently infiltrate tumors, modulate tumor angiogenesis, promote metastasis, and have been associated with tumor resistance to chemotherapy and immune checkpoint blockade (27, 28). A characteristic marker for myeloid cells is the immune checkpoint SIRPα. Therapeutic targeting the SIRPα/CD47 signaling axis is considered a promising strategy for the treatment of advanced cancers. Recent *in vivo* data have demonstrated a synergistic anti-tumor effect of SIRPα-specific antibodies in combination with tumor-opsonizing antibodies such as cetuximab (EGFR), rituximab (CD20), and trastuzumab human epidermal growth factor receptor (HER2) (25, 26, 29), and, currently, several anti-hSIRPα monoclonal antibodies including BI 765063 and GS-0189 (FIS-189) are in clinical trials for mono- and combination therapies (30). In addition to serving as therapeutic target, SIRPα also represents a biomarker, which can be used to stratify patients by myeloid cell expression patterns (17–19) and to track the migration and dynamics of myeloid cells in the context of cancer. Recently, murine-specific SIRPα Nbs were successfully employed for non-invasive single-photon emission tomography imaging of myeloid cells in intracranial glioblastoma tumors of experimental mice (31).

Here, we pursued a binary screening strategy to develop the first hSIRPα-specific Nbs as a panel of novel theranostic binding molecules. Our aim was either to identify Nbs as modulating biologics blocking the hSIRPα/hCD47 axis or to monitor TAMs as the most common myeloid cell type in the TME. By choosing Nbs that exclusively bind the D1 domain of hSIRPα, we were able to identify binders that selectively block the interaction with CD47 and enhance ADCP in combination with the tumor-opsonizing antibody cetuximab *in vitro*. In particular, the selectivity of Nb S45 for binding hSIRPα, but not hSIRPγ, might be advantageous, as recent data showed that nonselective hSIRPα/hSIRPγ blockade can impair T-cell activation, proliferation, and endothelial transmigration (32). Notably, as versatile building blocks, Nbs can easily be customized into more effective biologics. Thus, blocking efficacies of the inhibitory hSIRPα-specific Nbs can be further improved, e.g., by establishing bivalent or biparatopic formats as previously shown (24, 33). Alternatively, bispecific binding molecules could be generated, e.g., by fusing the hSIRPα-blocking Nbs with a tumor-opsonizing Nb and Fc moiety (34, 35) or CD40L expressed by activated T cells to bridge innate and adaptive immune responses (36). To address rapid renal clearance, which is a major drawback of small-sized Nbs for therapeutic application, other modifications such as PEGylation, addition of an albumin-binding moiety, or direct linkage to carrier proteins can be considered to extend their systemic half-life (t½) and efficacy (37, 38).

In addition to developing inhibitory hSIRPα Nbs, we also identified binders to elucidate the presence and infiltration of the myeloid cell population using PET-based non-invasive *in vivo* imaging. Current diagnostic methods are based on histology and thus require biopsies through invasive sampling or endpoint analyses. These methods can be associated with severe side effects and limit the predictive value of such diagnostic approaches. In contrast, non-invasive *in vivo* whole-body molecular imaging techniques, particularly PET, represent a powerful method to monitor and quantify specific cell populations and thereby support individual therapy decisions (39–41). Because of their ideal characteristics for PET imaging, including specific binding, fast tissue penetration, and rapid renal clearance, Nbs emerged as next-generation tracer molecules with numerous candidates in preclinical and first candidates in clinical testing (42–44). With the hSIRPα-S36 Nb, we selected a functionally inert but high-affinity binding candidate for which we achieved site-directed chemical chelator labeling based on a unique protein engineering approach that did not compromise the stability or binding properties. Compared with other, more elaborate and less effective labeling strategies such as sortagging (45–47), this approach resulted in rapid chelator conjugation by applying straightforward NCS chemistry.

^64^Cu-hSIRPα-S36_K>R_ Nb–PET/MR imaging in a novel tumor-bearing hSIRPα/hCD47 KI mouse model revealed rapid recruitment and sustained accumulation of our radiotracer in myeloid-enriched tumors and lymphatic organs with low background signal. We also observed a significantly enhanced ^64^Cu-hSIRPα-S36_K>R_ Nb uptake in MC38-hCD47 adenocarcinomas of hSIRPα/hCD47 KI mice vs. wt mice, suggesting specific targeting of myeloid cells within the TME. This is also supported by the fact that no enhanced ^64^Cu-hSIRPα-S36_K>R_ Nb uptake was observed in tumors and lymphatic organs of murine SIRPα and CD47 expressing wt mice. Beyond the crucial role of myeloid cells in tumor progression and cancer immunotherapy resistance, the occurrence of myeloid cells in diseased tissues is a hallmark of several inflammatory diseases like SARS-CoV-2 infection or autoimmune diseases such as systemic sclerosis, rheumatoid arthritis, and inflammatory bowel disease (48, 49). Thus, the non-invasive *in vivo* monitoring of biodistribution, density, and dynamic changes of the myeloid cell compartment presented in this initial study would allow surveillance and early assessment of therapeutic response in a variety of diseases (50). In comparison to established strategies typically targeting TAM subpopulations visualizing the Translocator protein (TSPO) or the macrophage mannose receptor (MMR) using the ^68^Ga anti-MMR Nb, the ^64^Cu-hSIRPα-S36_K>R_ Nb enables the monitoring of the entire myeloid cell population (11, 51, 52). Furthermore, given that hSIRPα-S36 Nb detects both hSIRPα allelic variants, its application is not restricted to patient subpopulations.

In summary, this study demonstrates for the first time the generation and detailed characterization of hSIRPα-specific Nbs for potential therapeutic and diagnostic applications. Considering the important role of myeloid cells, particularly TAMs, the herein developed hSIRPα-blocking Nbs have the potential to extend current macrophage-specific therapeutic strategies (30, 53). Moreover, our novel ^64^Cu-hSIRPα-S36_K>R_ Nb–based PET tracer will broaden the growing pipeline of Nb-based radiotracers to selectively visualize tumor-associated immune cells by non-invasive *in vivo* PET imaging (45, 47, 51, 54). Given the increasing importance of personalized medicine, we anticipate that the presented hSIRPα-specific Nbs might find widespread use as novel theranostics either integrated into or accompanying emerging immunotherapies.

## Materials and methods

### Nanobody screening

For the selection of hSIRPα-specific Nbs, two consecutive phage enrichment rounds either with immobilized hSIRPα or hSIRPαD1 were performed. To generate Nb-presenting phages, TG1 cells comprising the Nb-library in pHEN4 were infected with the M13K07 helper phage. In each panning round, 1 × 10^11^ phages were applied to streptavidin or neutravidin plates (Thermo Fisher Scientific) coated with biotinylated antigen (5 µg/mL). For biotinylation, purified antigen (Acrobiosystems) was reacted with Sulfo-NHS-LC-LC-Biotin (Thermo Fisher Scientific) in 5 M excess at ambient temperature for 30 min. Excess of biotin was removed by size exclusion chromatography using Zeba™ Spin Desalting Columns 7K MWCO 0.5 mL (Thermo Fisher Scientific) according to the manufacturer’s protocol. Blocking of antigen and phage was performed alternatively with 5% milk or Bovine Serum Albumin (BSA) in Phosphate-Buffered Saline with Tween (PBS-T), and, as the number of panning rounds increased, the wash stringency with PBS-T was intensified. Bound phages were eluted in 100 mM triethylamine (TEA) (pH 10.0), followed by immediate neutralization with 1 M Tris/HCl (pH 7.4). Exponentially growing TG1 cells were infected with eluted phages and spread on selection plates for subsequent selection rounds. In each round, antigen-specific enrichment was monitored by counting colony-forming units.

### Whole-cell phage ELISA

For the monoclonal phage enzyme linked immunosorbent assay (ELISA) individual clones were picked, and phage production was induced as described above. Moreover, 96-well cell culture plates (Corning) were coated with poly-L-lysine (Sigma-Aldrich) and washed once with H_2_O. U2OS-wt and U2OS overexpressing hSIRPα (U2OS-hSIRPα) or hSIRPαD1 (U2OS-hSIRPαD1) were plated at 2 × 10^4^ cells per well in 100 µL and grown overnight. The next day, 70 µL of phage supernatant was added to each cell type and incubated at 4°C for 3 h. Cells were washed five times with 5% FBS in PBS, followed by adding the Anti-M13 Monoclonal Antibody coulpled Horseradish Peroxidase (M13-HRP)–labeled detection antibody (Progen, 1:2,000 dilution) for 1 h, and washed three times with 5% Fetal Bovine Serum (FBS) in PBS. Finally, Onestep ultra TMB 32048 ELISA substrate (Thermo Fisher Scientific) was added to each well and incubated until color change was visible before stopping the reaction with 100 µL of 1 M H_2_SO_4_. For detection, the Pherastar plate reader at 450 nm was applied, and phage ELISA-positive clones were defined by a two-fold signal above wt control cells.

### Protein expression and purification

hSIRPα Nbs were cloned into the pHEN6 vector (55) and expressed in XL-1 as previously described (22, 56). Sortase A pentamutant (eSrtA) in pET29 was a gift from David Liu (Addgene, plasmid # 75144) and was expressed as published (57). Expressed proteins were purified by immobilized metal affinity chromatography (IMAC) using a HisTrap^FF^ column followed by a size exclusion chromatography (SEC; Superdex 75) on an Aekta pure system (Cytiva). Quality of all purified proteins was analyzed via standard Sodium Dodecyl Sulfate – Polyacrylamid Gel Electrophoresis (SDS-PAGE) under denaturizing conditions [5 min, 95°C in 2× SDS-sample buffer containing 60 mM Tris/HCl (pH 6.8); 2% (w/v) SDS; 5% (v/v) 2-mercaptoethanol, 10% (v/v) glycerol, 0.02% bromphenole blue]. For protein visualization, InstantBlue Coomassie (Expedeon) staining or alternatively immunoblotting as previously published (58) was performed. Protein concentration was determined by NanoDrop ND100 spectrophotometer.

### Biolayer interferometry

Analysis of binding kinetics of hSIRPα-specific Nbs was performed using the Octet RED96e system (Sartorius) as per the manufacturer’s recommendations. In brief, biotinylated hSIRPα (5 µg/mL) diluted in Octet buffer (PBS, 0.1% BSA, and 0.02% Tween-20) was immobilized on streptavidin coated biosensor tips (SA, Sartorius) for 40 s. In the association step, a dilution series of Nbs ranging from 0.625 nM to 320 nM were reacted for 240 s followed by dissociation in Octet buffer for 720 s. Every run was normalized to a reference run applying Octet buffer for association. Data were analyzed using the Octet Data Analysis HT 12.0 software applying the 1:1 ligand-binding model and global fitting. For epitope binning, two consecutive association steps with different Nbs were performed. By analyzing the binding behavior of the second Nb, conclusions about shared epitopes were drawn. For the hCD47 competition assay, hCD47 was biotinylated and immobilized on SA biosensors followed by the application of pre-mixed solutions containing hSIRPα (20 nM) and Nb (250 nM). hCD47-competing Ab KWAR23 (5 nM) was used as control.

### Live-cell immunofluorescence

Stably expressing hSIRPα U2OS cells, U2OS wt or U2OS cells transiently expressing individual hSIRPα domains (D1-3) with SPOT-Tag, or different hSIRP family members (hSIRPα-V1, hSIRPα-V2, hSIRPβ1, hSIRPy, and murine SIRPα) were plated at ~10,000 cells per well of a µClear 96-well plate (Greiner Bio One, cat. #655090) and cultivated overnight in standard conditions. For imaging, medium was replaced by live-cell visualization medium DMEMgfp-2 (Evrogen, cat. #MC102) supplemented with 10% FBS, 2 mM L-glutamine, Hoechst33258 (2 µg/mL; Sigma-Aldrich) for nuclear staining. Unlabeled hSIRPα Nbs (1 nM to 100 nM) in combination with anti-VHH secondary Cy5 AffiniPure Goat Anti-Alpaca IgG (2.5 µg/mL; Jackson Immuno Research) were added and incubated for 1 h at 37°C. For control staining, hSIRPα Ab Phycoerythrin (PE) (SE5A5, BioLegend) and bivSPOT-Nb labeled with AlexaFluor647 (AF647) were used. Images were acquired with a MetaXpress Micro XL system (Molecular Devices) at ×20 or ×40 magnification.

### Stability analysis

Stability analysis was performed by the Prometheus NT.48 (Nanotemper). In brief, freshly thawed hSIRPα Nbs were diluted to 0.25 mg/mL, and measurements were carried out at time point T_0_ or after incubation for 10 days at 37°C (T_10_) using high-sensitivity capillaries. Thermal unfolding and aggregation of the Nbs were induced by the application of a thermal ramp of 20°C to 95°C while measuring fluorescence ratios (F350/F330) and light scattering. Via the PR. ThermControl v2.0.4, the melting temperature (T_M_) and aggregation (T_Agg_) temperature were determined.

### Fluorescent labeling

For sortase coupling, 50 μM Nb, 250 μM sortase peptide (H-Gly-Gly-Gly-propyl-azide synthesized by Intavis AG) dissolved in sortase buffer [50 mM Tris (pH 7.5) and 150 mM NaCl], and 10 μM sortase were mixed in coupling buffer (sortase buffer with 10 mM CaCl_2_) and incubated for 4 h at 4°C. To stop the reaction and remove uncoupled Nb and sortase, an IMAC was performed, followed by protein concentration, and unreacted sortase peptide depletion using the Amicon Ultra-Centrifugal Filter 3-kDa MWCO. For fluorescent labeling, the SPAAC (strain-promoted azide-alkyne cycloaddition) click chemistry reaction was employed by incubating azide-coupled Nbs with two-fold molar excess of DBCO-AF647 (Jena Bioscience) for 2 h at 25°C. Excess DBCO-AF647 was subsequently removed by dialysis (GeBAflex-tube, 6–8 kDa, Scienova). Finally, a hydrophobic interaction chromatography (HiTrap Butyl-S FF, Cytiva) was performed to deplete unlabeled Nb.

### PBMC isolation, cell freezing, and thawing

Fresh blood, buffy coats, or mononuclear blood cell concentrates were obtained from healthy volunteers at the Department of Immunology or from the ZKT Tübingen gGmbH. Participants gave informed written consent, and the studies were approved by the ethical review committee of the University of Tübingen, projects 156/2012B01 and 713/2018BO2. Blood products were diluted with PBS 1× (homemade from 10× stock solution, Lonza, Switzerland), and PBMCs were isolated by density gradient centrifugation with Biocoll separation solution (Biochrom, Germany). PBMCs were washed twice with PBS 1×, counted with a NC-250 cell counter (Chemometec, Denmark), and resuspended in heat-inactivated (h.i.) fetal bovine serum (Capricorn Scientific, Germany) containing 10% Dimethylsulfoxide (DMSO) (Merck). Cells were immediately transferred into a −80°C freezer in a freezing container (Mr. Frosty; Thermo Fisher Scientific). After at least 24 h, frozen cells were transferred into a liquid nitrogen tank and were kept frozen until use. For the experiments, cells were thawed in Iscove's Modified Dulbecco's Medium (IMDM) (+L-Glutamin + 25 mM (4-(2-hydroxyethyl)-1-piperazineethanesulfonic acid) HEPES; Life Technologies) supplemented with 2.5% h.i. human serum (HS; PanBiotech, Germany), 1× Penicillin-Streptomycin (P/S) (Sigma-Aldrich), and 50 µm β-Mercaptoethanol (Merck), washed once, counted, and used for downstream assays.

### Flow cytometry

For flow cytometry analysis, ~200,000 cells per staining condition were used in flow cytometry buffer: PBS containing 0.02% sodium azide, 2 mM EDTA, and 2% (v/v) FBS (Thermo Fisher Scientific). Extracellular staining was performed with hSIRPα Nbs conjugated to AF647 (200 nM), CD3 Ab Allophycocyanin- Cyanine 7 (APC/Cy7) (HIT3a, BioLegend), CD14 Ab PE (HCD14, BioLegend), dead cell marker Zombie Violet (BioLegend) or the respective unspecific fluorescently labeled Pep Nb (PEP-Nb_AF647_) (58), the positive control hSIRPα Ab PE (SE5A5, BioLegend), and isotype control Abs (BioLegend), by incubation for 45 min at 4°C. Cells were washed three times with Fluorescence Activated Cell Sorting/ Flow Cytometry (FACS) buffer, and data were acquired on the same day using an LSRFortessa™ flow cytometer (Becton Dickinson) equipped with the DIVA Software (Becton Dickinson). Final data analysis was performed using the FlowJo10® software (Becton Dickinson).

### Macrophage-mediated antibody-dependent cellular phagocytosis assay

CD14^+^ cells were purified from frozen PBMCs and CD14-positive selection (Miltenyi Biotec) according to the manufacturer’s protocols. MDMs were generated by seeding three million CD14^+^ cells into one six-well plate (Nunc™, Thermo Fisher Scientific) in IMDM (Thermo Fisher Scientific) supplemented with 10% (v/v) fetal bovine serum (Thermo Fisher Scientific) and M-CSF (50 ng/mL; Miltenyi Biotec) and cultured for 7 to 9 days. Cells were detached from culture plates with Accutase® (Sigma-Aldrich). DLD-1 cells were labeled with the CFSE Cell Division Tracker Kit (BioLegend) according to manufacturer’s instructions. A total of 100,000 DLD-1 cells and 50,000 MDMs were incubated in U-bottom 96-well plates (Corning) with hSIRPα Nbs (1 µM) or KWAR23 (100 nM) and cetuximab (0.66 nM) (MedChemExpress) for 2 h at 37° C, followed by detachment of adherent cells from culture plates with Accutase® (Sigma-Aldrich). For flow cytometry, cells were incubated with CD206 Ab AF647 (clone 15–2, BioLegend) and dead cell marker Zombie Violet (BioLegend). Percent of phagocytosis indicates the percentage of viable CD206^+^CFSE^+^ macrophages.

### Chelator conjugation and radiolabeling

For chelator conjugation and radiolabeling with ^64^Cu, metal-free equipment and buffers pretreated with Chelex 100 (Sigma-Aldrich) were used. Nbs (100 µg) were reacted with 100 M equivalents of p-NCS-benzyl-NODA-GA (CheMatech) in 0.2 M sodium bicarbonate (pH 8.7) for 24 h at room temperature (RT). Excess of chelator was removed by ultrafiltration (Amicon Ultra 0.5 mL, 3-kDa MWCO, Merck Millipore) using the same buffer conditions. For neutralization of [^64^Cu]CuCl_2_ (300 MBq in 0.1 M HCl), 1.5 volumes of 0.5 M ammonium acetate solution (pH 4.1) were added, resulting in a pH of 4. Conjugate (150 µg) was added to the solution and incubated at 35°C for 30 min. A 0.2% diethylenetriaminepentaacetic acid (3 µL) solution was added to quench the labeling reaction. Complete incorporation of the radioisotope was confirmed after each radiosynthesis by thin-layer chromatography [Agilent Technologies; mobile phase, 0.1 M sodium citrate buffer (pH 5)] and high-performance size exclusion chromatography (Superdex 75 Increase, 300 × 10 mm, Cytiva; mobile phase, DPBS with 0.5 mM EDTA, adjusted to pH 6.9).

### *In vitro* radioimmunoassay

To determine the immunoreactive fraction (maximum binding, B_max_), an increasing number of HT1080-hSIRPα cells were incubated in triplicates with 1 ng (2 MBq/µg) of ^64^Cu-hSIRPα-S36_K>R_ Nb for 1 h at 37°C and washed twice with PBS/1% FBS. The remaining cell-bound radioactivity was measured using a Wizard² 2480 gamma counter (PerkinElmer Inc.) and quantified as percentage of the total added activity.

### Tumor-bearing mouse models and PET imaging

Six-week-old female C57BL/6N wt mice were purchased from Charles River. C57BL/6 hSIRPα/hCD47 KI (C57BL/6N^CD47tm1.1(CD47)Geno;Sirpαtm2.1(SIRPA)Geno^) mice (hSIRPα/hCD47) were developed by genOway (manuscript in preparation). For tumor cell inoculation, 1 × 10^6^ MC38-hPD-L1-hCD47-luciferase-Zsgreen (MC38-hCD47) KI colon adenocarcinoma cells (developed by genOway) were resuspended in 100 µL of PBS and subcutaneously injected into hSIRPα/hCD47or wt mice.

hSIRPα/hCD47 and wt mice were injected intravenously (*i.v.*) with 5 µg (~10 MBq) of ^64^Cu-hSIRPα-S36_K>R_ Nb or ^64^Cu-GFP_K>R_ Nb 9 days after tumor cell inoculation. Mice were anesthetized with 1.5% isoflurane in 100% oxygen during the scans. Ten-minute static PET scans were performed after 5 min, 90 min, 3 h, and 6 h in a dedicated small-animal Inveon microPET scanner (Siemens Healthineers) with temperature-controlled heating mats. For anatomical colocalization, sequential T2 TurboRARE MR images were acquired immediately after the PET scans on a small animal 7 T ClinScan magnetic resonance scanner (Bruker BioSpin GmbH). PET images were reconstructed using an ordered subset expectation maximization (OSEM3D) algorithm and analyzed with Inveon Research Workplace (Siemens Preclinical Solutions). The volumes of interest of each organ were defined on the basis of anatomical MRI to acquire the corresponding PET tracer uptake within the tumor and organs of interest. The resulting radioactive concentration was measured per tissue volume (Becquerel/cubic centimeter) decay-corrected and presented as percentage of injected dose per cubic centimeter (%ID/cc).

### Analyses, statistics, and graphical illustrations

Graph preparation and statistical analysis were performed using the GraphPad Prism Software (version 9.0.0 or higher). One-way ANOVA was performed for multiple comparisons using Tukey as a *post-hoc* test (mean and SEM). A value of p < 0.05 was considered statistically significant and marked as * for p < 0.05, ** for p < 0.01, *** for p < 0.001, and **** for p < 0.0001; non-significant results were marked with ns. Graphical illustrations were created with BioRender.com.

## Data availability statement

The original contributions presented in the study are included in the article/**Supplementary Material**. Further inquiries can be directed to the corresponding author.

## Ethics statement

The studies involving humans were approved by the ethical review committee of the University of Tübingen, projects 156/2012B01 and 713/2018BO2. The studies were conducted in accordance with the local legislation and institutional requirements. The participants provided their written informed consent to participate in this study.

## Author contributions

TW: Investigation, Methodology, Writing – original draft, Writing – review & editing. SB: Investigation, Methodology, Writing – original draft. IL: Investigation, Writing – original draft. DF: Investigation, Writing – original draft. MG: Investigation, Methodology, Writing – original draft. BT: Investigation, Methodology, Writing – original draft. PK: Investigation, Methodology, Writing – original draft. DSe: Investigation, Writing – original draft. SM: Methodology, Writing – original draft. AR: Resources, Writing – original draft. FS: Resources, Writing – original draft. KT: Resources, Writing – original draft. SP: Investigation, Methodology, Writing – original draft. AZ: Resources, Writing – original draft. CG: Resources, Writing – original draft. AS: Resources, Writing – original draft. SN: Resources, Writing – original draft. AM: Investigation, Writing – original draft. MK: Conceptualization, Writing – original draft. BP: Conceptualization, Formal analysis, Resources, Writing – original draft. DSo: Investigation, Writing – original draft, Writing – review & editing. UR: Conceptualization, Funding acquisition, Investigation, Supervision, Writing – original draft, Writing – review & editing.
