# Supplementary material for: Two birds with one stone: human SIRPα nanobodies for functional modulation and in vivo imaging of myeloid cells
Source: Front Immunol. 2023 Dec 18;14:1264179. doi: 10.3389/fimmu.2023.1264179 (PMC10757926; doi:10.3389/fimmu.2023.1264179)
Supplement: Supplementary file 1 [file DataSheet_1.pdf]

Supplementary Information

Two birds with one stone: human SIRPα nanobodies for functional modulation and in vivo imaging of myeloid cells

Teresa R. Wagner[1,2], Simone Blaess[3], Inga B. Leske[2], Desiree I. Frecot[1,2], Marius Gramlich[1], Bjoern Traenkle[1], Philipp D. Kaiser[1], Dominik Seyfried[3,4], Sandra Maier[1], Amélie Rezza[5], Fabiane Sônego[5], Kader Thiam[5], Stefania Pezzana[3], Anne Zeck[1], Cécile Gouttefangeas[4,6,7], Armin M. Scholz[8], Stefan Nueske[8], Andreas Maurer[3,7], Manfred Kneilling[3,7,9], Bernd J. Pichle[3,4,7], Dominik Sonanini[3,10], Ulrich Rothbauer[2,7#]

Affiliations

[1] NMI Natural and Medical Sciences Institute at the University of Tübingen, Reutlingen, Germany

[2] Pharmaceutical Biotechnology, Eberhard Karls University Tübingen, Germany

[3] Werner Siemens Imaging Center, Department of Preclinical Imaging and Radiopharmacy, University of Tübingen, Tübingen, Germany

[4] German Cancer Consortium (DKTK) and German Cancer Research Center (DKFZ) partner site Tübingen, Tübingen, Germany

[5] Preclinical Models & Services, genOway, Lyon, France

[6] Department of Immunology, Institute of Cell Biology, University of Tübingen, Tübingen, Germany

[7] Cluster of Excellence iFIT (EXC2180) "Image-Guided and Functionally Instructed Tumor Therapies", University of Tübingen, Germany

[8] Livestock Center of the Faculty of Veterinary Medicine, Ludwig Maximilians University Munich, Oberschleissheim, Germany

[9] Department of Dermatology, University of Tübingen, Tübingen, Germany

[10] Department of Medical Oncology and Pneumology, University of Tübingen, Tübingen, Germany

# corresponding author

Prof. Dr. Ulrich Rothbauer, Pharmaceutical Biotechnology, Eberhard Karls University Tübingen, Auf der Morgenstelle 8, 72076 Tübingen, Germany.

E-mail: ulrich.rothbauer@uni-tuebingen.de

Phone: +49 7071 29 72469

Fax: +49 7071 29-2476

Orcid ID: 0000-0001-5923-8986

Supplementary Materials & Methods

Expression constructs

DNA coding for hSIRPαV1 (GenBank accession: NM_001040022.1) and hSIRPαV2 (GenBank accession: D86043.1) were synthesized and cloned into NheI and EcoRI site of pcDNA3.1(+) (GenScript Biotech). The vector backbone was adapted by cutting with EcoRI and BstBI and insertion of DNA comprising an internal ribosomal entry site (IRES) and genes for GFP and Blasticidin S deaminase from the expression construct described in (1). For the generation of hSIRPα expression constructs comprising Ig-like V-type domain (D1, aa 31-146), Ig-like C1-type 1 (D2, aa147-252) and Ig-like C1-type 2 (D3, aa253-348), of UniProtKB P13987 were genetically fused N-terminally to aa1-26 of huCD59 (UniProtKB P13987) and SPOT-Tag (2) and C-terminally to aa91-128 of huCD59 and cloned into BglII and NotI sites of pEGFPN2 expression vector. huCD59 sequences of the expressed fusion protein causes both translocation to the endoplasmatic reticulum and GPI anchoring of the protein at the plasma membrane. DNA encoding for hSIRPβ and hSIRPγ were purchased from addgene (Plasmid #116790) (3) and Sino Biological (Catalog Number HG16111-NH) and subcloned into NheI and EcoRI sites of expression vector used for hSIRPα variants. Expression vector for murine SIRPα was generated based on reference sequence NM_007547.4 and includes SPOT-Tag subsequent to signal peptide (aa 1-31) (2). To generate the respective expression construct, cDNA was cloned into KpnI and XbaI restriction sites of pCMV3-C-FLAG vector.

Cell culture, transfection, stable cell line generation

U2OS, DLD-1, and HT-1080 cells (ATCC) were cultivated according to standard protocols in media containing DMEM (Thermo Fisher Scientific) or RPMI (Thermo Fisher Scientific), respectively supplemented with 10% (v/v) FBS (Thermo Fisher Scientific) and penicillin/streptomycin (Thermo Fisher Scientific) at 37°C and 5% CO_2 atmosphere in a humidified chamber and passaged using 0.05% trypsin-EDTA (Thermo Fisher Scientific). For transfection, Lipofectamine 2000 (Thermo Fisher Scientific) was used according to the manufacturer's protocol. To generate cells stably expressing hSIRPα, selection pressure was applied 24 h after transfection with 5 µg/ml Blasticidin S (Sigma Aldrich) for a period of two

weeks, followed by single cell separation. Finally, individual clones were analyzed for hSIRPα expression.

Cell Isolation

PBMCs were isolated as described previously (1). In brief, fresh blood was obtained from healthy volunteers and PBMCs were isolated by density gradient centrifugation with Biocoll separation solution (Biochrom) and frozen in heat-inactivated FBS (Capricorn Scientific, Germany) containing 10% dimethyl sulfoxide (DMSO; Merck).

Nanobody library generation

For alpaca immunization and Nb library generation, a similar protocol as previously described was performed (4, 5). Briefly, two alpacas (*Vicugna pacos*) were immunized with the extracellular portion of hSIRPα (aa31-370) produced in HEK293 cells (Acrobiosystems) with the approval of the Government of Upper Bavaria (approval number: 55.2-1-54-2532.0-80-14). After an initial vaccination with 560 µg, animals received five boost injections of 280 µg hSIRPα every two weeks. Finally, 91 days after initial immunization, ~100 ml of blood was collected, and lymphocytes were isolated by Ficoll gradient centrifugation with lymphocyte separation medium (PAA Laboratories GmbH). To obtain cDNA, total RNA was extracted using TRIzol (Life Technologies), followed by mRNA transcription using the First-Strand cDNA Synthesis Kit (GE Healthcare). The Nb repertoire was isolated and amplified in three subsequent PCR reactions using the following primer combinations: (1) CALL001 and CALL002, (2) forward primers FR1-1, FR1-2, FR1-3, FR1-4, and reverse primer CALL002, and (3) forward primers FR1-ext1 and FR1-ext2 and reverse primers FR4-1, FR4-2, FR4-3, FR4-4, FR4-5, and FR4-6 introducing SfiI and NotI restriction sites (1). Finally, the amplified Nb library was cloned into the pHEN4 phagemid vector (6) using the SfiI/NotI sites.

Hydrogen-deuterium exchange

HDX-MS epitope mapping was performed as recently described (7). In brief, 5 µL hSIRPα (42 µM) was incubated with either 2.5 µL PBS or a specific hSIRPα Nb S8 (103 µM), S33 (145 µM) or S36 (78 µM). After a 10 min pre-incubation at 25 °C, HDX was initiated by a 1:10 dilution in PBS (pH 7.4) prepared with D_2O (final labeling D_2O concentration = 90%). Aliquots of 15 µL

were quenched after deuteration for 5 and 30 min by adding 15 µL ice-cold quenching solution (200 mM TCEP, 1.5% formic acid and 4 M guanidine HCl in 100 mM ammonium formate solution, pH 2.2), resulting in a final pH of 2.5. Samples were immediately snap frozen and stored at -80 °C until analysis. Non-deuterated control samples were processed using PBS prepared with H_2O. Each sample was prepared in independent technical replicates (n=3). Settled gel of immobilized pepsin (Thermo Fisher Scientific) was prepared by centrifugation of 60 µL 50% slurry (in ammonium formate solution pH 2.5) at 1,000 x g and 0 °C for 3 min. The supernatant was discarded, sample aliquots were thawed and added to the settled pepsin gel. The proteolysis was performed for 2 min in an ice-water bath. To improve sequence coverage near the N-glycosylation sites of hSIRPα, a post-proteolysis deglycosylation was performed using PNGase Rc (7). 5 µL PNGase Rc (4 µM) was added under a filter inlet (0.65 µm, Merck Millipore) and the proteolyzed sample was placed on the filter. Centrifugation at 1000 x g for 30 s at 0 °C removed the beads and initiated the deglycosylation of the peptides in the flow-through. Deglycosylation was carried out in an ice-water bath for an additional 2 min, and samples were analyzed by LC-MS as described in (8).

Data analysis was performed as previously described in (8). HDX data were obtained for ≥83% of the hSIRPα sequence. The deuterium uptake of each peptide was normalized to the maximal exchangeable protons of the backbone. The deuteration was compared between hSIRPα alone and in complex. A peptide was considered protected from HDX if the summed difference was ≥5%. A peptide was considered not protected if the summed HDX difference was ≤3%.

Mass spectrometry

To confirm correct expression, integrity and purity, chelator conjugated hSIRPα-S36$_{K>R}$ was analyzed by mass spectrometry. Protein sample (5 µg) was diluted 1:3 with HisNaCl buffer (20 mM His, 140 mM NaCl, pH 6.0) and analyzed by liquid chromatography (HPLC) coupled to electrospray ionization (ESI) quadrupole time-of-flight (QTOF) MS. Sample (0.4 µg per injection) was desalted using reversed phase chromatography on a Dionex U3000 RSLC system (Thermo Scientific, Dreieich, Germany) using a Acquity BEH300 C4 column (1mm x

50mm, Waters, Eschborn, Germany) at 75°C and 150 µl/min flow rate applying a 11-min linear gradient with varying slopes. In detail, the gradient steps were applied as follows (min/% Eluent B): 0/5, 0.4/5, 2.55/30, 7/50, 7.5/99, 8/5, 8.75/99, 9.5/5, 10/99, 10.25/5 and 11/5. Eluent B was acetonitrile with 0.1% formic acid, and solvent A was water with 0.1% formic acid. To avoid contamination of the mass spectrometer with buffer salts, the HPLC eluate was directed into waste for the first 2 min. Continuous MS analysis was performed using a QTOF mass spectrometer (Maxis UHR-TOF; Bruker, Bremen, Germany) with an ESI source operating in positive ion mode. Spectra were taken in the mass range of 600–2000 m/z. External calibration was applied by infusion of tune mix via a syringe pump during a short time segment at the beginning of the run. Raw MS data were lock-mass corrected (at m/z 1221.9906) and further processed using Data Analysis 5.3 and MaxEnt Deconvolution software tools (Bruker).

Supplementary Tables

Supplementary Table 1. Amino acid sequences of hSIRPα Nbs.

hSIRPα Nb	Amino acid sequence
**S7**	HVQLVESGGGLVQVGDSLRLSCAPSGRTFRAYAMAWFRQAPGKEREFVAAIRWIGGTPSYK<br>DSVKGRFTISRDNARNTFYLQMNSLEPQDTGVYYCAAEETQKGSGLGHDGRHYDYWGQGT<br>PVTVSS
**S8**	QVQLVESGGGLVQPGGSLRLSCADSEFRLDEYATGWFRQAPGKEREGVACISRSGSNTKYT<br>DSVKGRFTISRDNAKKTVYLQMNSLKPEDTAVYYCAADLTSHYLSCSSYTDYNSWGQGTQVT<br>VSS
**S12**	QVQLVESGGGLVQPGGSLRLSCLVSGFSSSYYAIGWFRQAPGKEREGVSCITRSGDTTNYAD<br>SVKGRFTISRDDARNTVYLQMNSLKTEDTAVYSCALWAWAGSGRLRCTASEYGHWGQGTQ<br>VTVSS
**S14**	EVQLVESGGGLVQPGGSLRLSCAASGFTANSYRMTWVRQAPGKGLEWVADIGTGNEWKYY<br>PDSVKGRFTISRDAAKNTVYLQMNSLKPEDTAVYYCAKNGAVWYGEDGLDYWGKGTLVTVS<br>S
**S17**	QVQLVESGGGLVQPGGSLRLSCAASGFTLDSYVIGWFRQAPGKEREGVSCISQRIGSTDYAD<br>SVKGRFTISKDNAKNTAYLQMNALKPEDTAVYYCAAGRLYYSGTYCPQGGMSYWGKGSPVT<br>VSS
**S21**	DVQLVESGGGLVQPGGSLTLSCAASGFTSGTYALAYFRQAPGKEREGVSCISNDGGSRNYA<br>DSVKGRFTISRDNAKNTMYLQMNSLKPEDTAVYYCAAGPWYYCSDFARRPDGMDYWGKGT<br>PVTVSS
**S29**	EVQLVESGGGLVQPGGSLRLSCAASGFTSETYAIGWFRQAPGKEREGVSCISNDGGSRNYA<br>DSVKGRFTISRDNAKNTSYLQMNSLKPEDTAVYYCAAGKYYYCLGYVSDGMDYWGKGTPVT<br>VSS
**S33**	EVQLVESGGGLVQPGGSLRLSCTTSGFTLDYYSVGWFRQAPGKEREGVSCITASGSSTNYA<br>NSVKGRFTISRDKVKNTVYLQMSSLKPEDTAVYYCAAEPCTVVAGILQAPTSDFGSWGQGTQ<br>VTVSS
**S36**	EVQLVESGGGLVQPGGSLRLSCAASGFTLDYYAIGWFRQVPGKEREGVSCISSDGGGNTYY<br>ADSVKGRFTISRDNAKNTVYLQMNSLKPEDSAVYSCAAGPTYYSGSYCRDPHSDYDYWGQG<br>TQVTVSS
**S41**	HVQLVESGGGLVQPGGSLRLSCAASGETLDYYAIGYVRQAPGKEREGVSCISSRGDNTNYAD<br>SVKGRFTISRDNANNTVYLQMNSLKPEDTAVYYCVAIKDSWQNYYCSVGWVEYDVWGQGTQ<br>VTVSS

**S42**	HVQLVESGGGLVQPGGSLRLSCAASGFIFSGYAMSWVRQAPGKGLEWVSDISSDGVRLYYA DSVKGRFTISRDNAKNTVYLEMTSLKPDDTALYYCATSSDPQLGLDLWGKGTPVTVSS
**S43**	HVQLVESGGGLVQPGGSARLSCAASGFDLDYFAIGWFRQAPGKEREGVSCISTRSETTNYVD SVKGRFTISRDNAISTVYLQMNNLKPEDTAVYYCAADWADWTMTGCQMTWDDYNYWGQGA QVTVSS
**S44**	QVQLQESGGGLVQPGGSLRLACAASGFTFSSYDMSWVRQAPGKGLEWVSDIKSGGGRTYY ADSVKGRFTISRDNAKNTVYLQMNSLKPEDTAVYYCGSLDNSGAYTGGGQGSQVTVSS
**S45**	HVQLVESGGGLVQPGGSLRLSCAARGFSFRDSAMSWARQAPGKGLEWVGAIYSDGSTVYE KSVKGRFTISRDNAKNTMYLQMHSLKPEDSARYYCAPKGVVADEGDYSGQGTLVTVSS

Supplementary Table 2. Summary of HDX-MS parameters of epitope mapping of anti-SIRPα-Nbs as per consensus guidelines (9).

**HDX parameters**			
States	SIRPα & SIRPα bound by **Nb S8**	SIRPα & SIRPα bound by **Nb S33**	SIRPα & SIRPα bound by **Nb S36**
HDX reaction detail	1x PBS pH 7.4, 25 °C, 90% D_2O	1x PBS pH 7.4, 25 °C, 90% D_2O	1x PBS pH 7.4, 25 °C, 90% D_2O
Deuteration time points	5 & 30 min	5 & 30 min	5 & 30 min
Average peptide length (AA)	14.2 (s_d = 6.6)	14.5 (s_d = 7.1)	14.3 (s_d = 6.7)
Average redundancy (AA)	4.4	4.4	4.3
Number of used peptides	108	106	105
Sequence coverage	86%	86%	86%
$\Delta\overline{HX}$ threshold for each time point (p≤0.05)	0.27 Da	0.21 Da	0.23 Da
$\Delta\overline{HX}$ threshold for each time point (p≤0.01)	0.44 Da	0.34 Da	0.38 Da
Complexed SIRPα during labelling	99.9%	99.0%	97.1%

Supplementary Figures

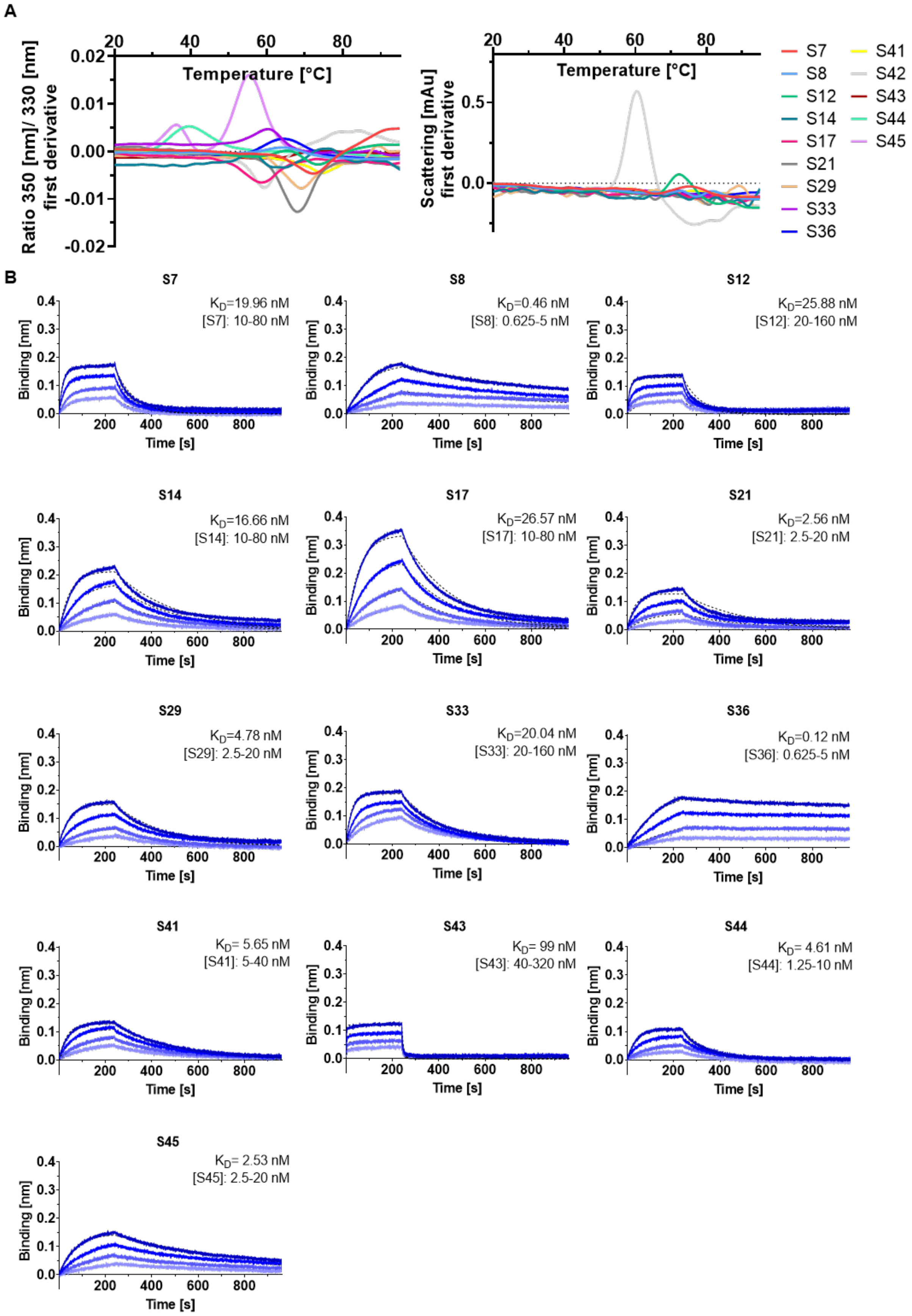

Supplementary Figure 1. Detailed stability analysis and kinetic measurements of hSIRPα Nbs.

A Stability of hSIRPα Nbs was analyzed by nano-differential scanning fluorimetry (nanoDSF). Fluorescence ratios (350 nm/330 nm) and light intensity loss due to scattering illustrated as first derivative are shown. Data are shown as mean value of three technical replicates.

B Sensograms of biolayer interferometry- (BLI-) based affinity measurements of 13 identified hSIRPα Nbs. Biotinylated hSIRPα was immobilized on streptavidin biosensors and kinetic measurements were performed by using four concentrations of purified Nbs ranging from 0.625 to 320 nM (displayed with gradually lighter shades of color). Binding affinity (K_D) was calculated from global 1:1 fits illustrated as dashed lines.

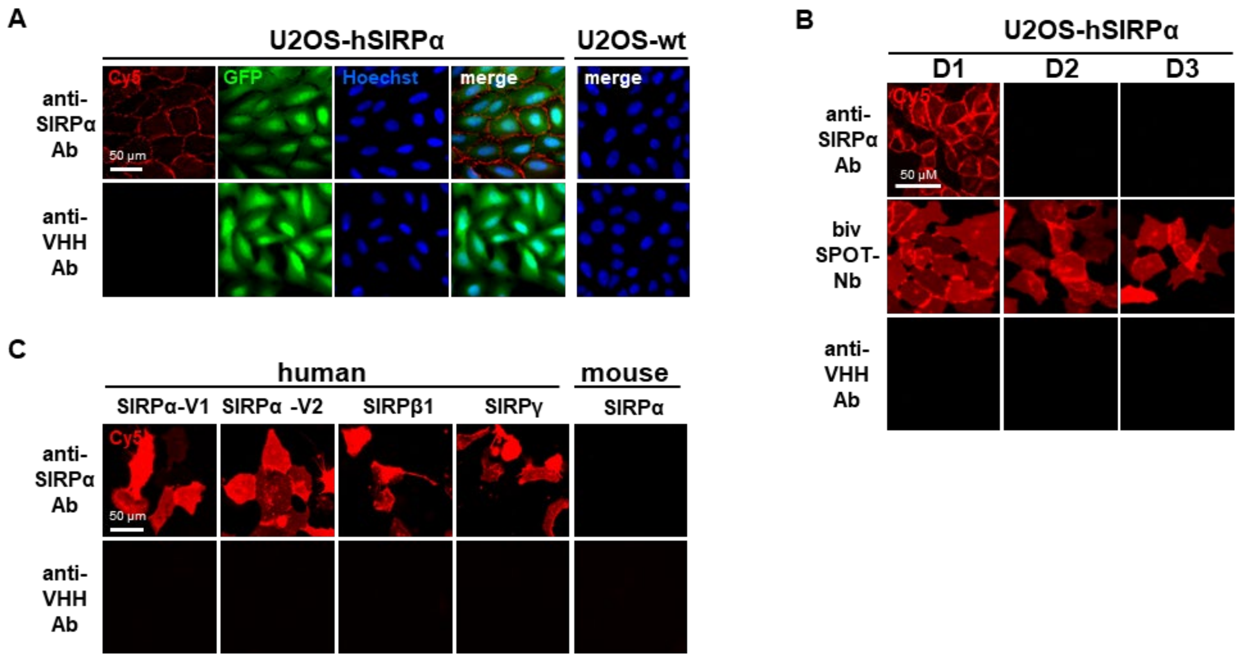

Supplementary Figure 2. Immunofluorescence staining controls.

A Immunofluorescence staining controls of U2OS cells displaying hSIRPα on their surface. Representative images of three technical replicates show hSIRPα Ab (SE5A5) and secondary only Ab control (anti-VHH-Cy5) (red), intracellular IRES derived GFP signal (green), nuclei staining (Hoechst, blue) and merged signals; scale bar: 50 µm.

B Immunofluorescence staining controls of U2OS cells displaying SPOT-tagged hSIRPα domain 1 (D1), domain 2 (D2) or domain 3 (D3) on their surface. Representative images of three technical replicates of live cells stained with hSIRPα Ab (SE5A5), bivSPOT-Nb (2) and secondary only Ab control (anti-VHH-Cy5) are shown; scale bar: 50 µm.

C Immunofluorescence control staining of U2OS cells expressing human hSIRPα-V1, -V2, hSIRPβ1, hSIRPγ or mouse hSIRPα on their surface. Representative images of three technical replicates of live cells stained with hSIRPα Ab (SE5A5) and secondary only Ab control (anti-VHH) are shown; scale bar: 50 µm.

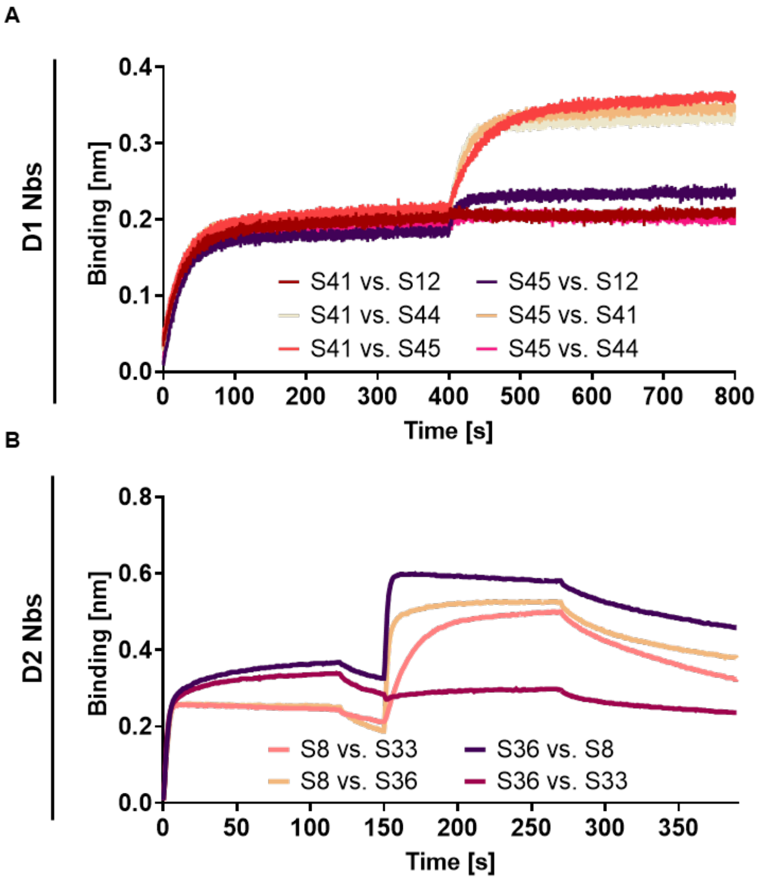

Supplementary Figure 3. **Epitope binning analysis of hSIRPα Nbs by BLI.**

A Sensograms of BLI-based epitope binning analysis of hSIRPαD1 Nbs are shown (n=1). Biotinylated hSIRPα was immobilized on streptavidin biosensors followed by two consecutive association steps of hSIRPαD1 Nbs S12, S41, S44, S45 (100 nM).

B Sensograms of BLI-based epitope binning analysis of hSIRPαD2 Nbs are shown (n=1). Biotinylated hSIRPα was immobilized on streptavidin biosensors followed by two consecutive association steps of hSIRPαD2 Nbs S8, S33, S36 (100 nM).

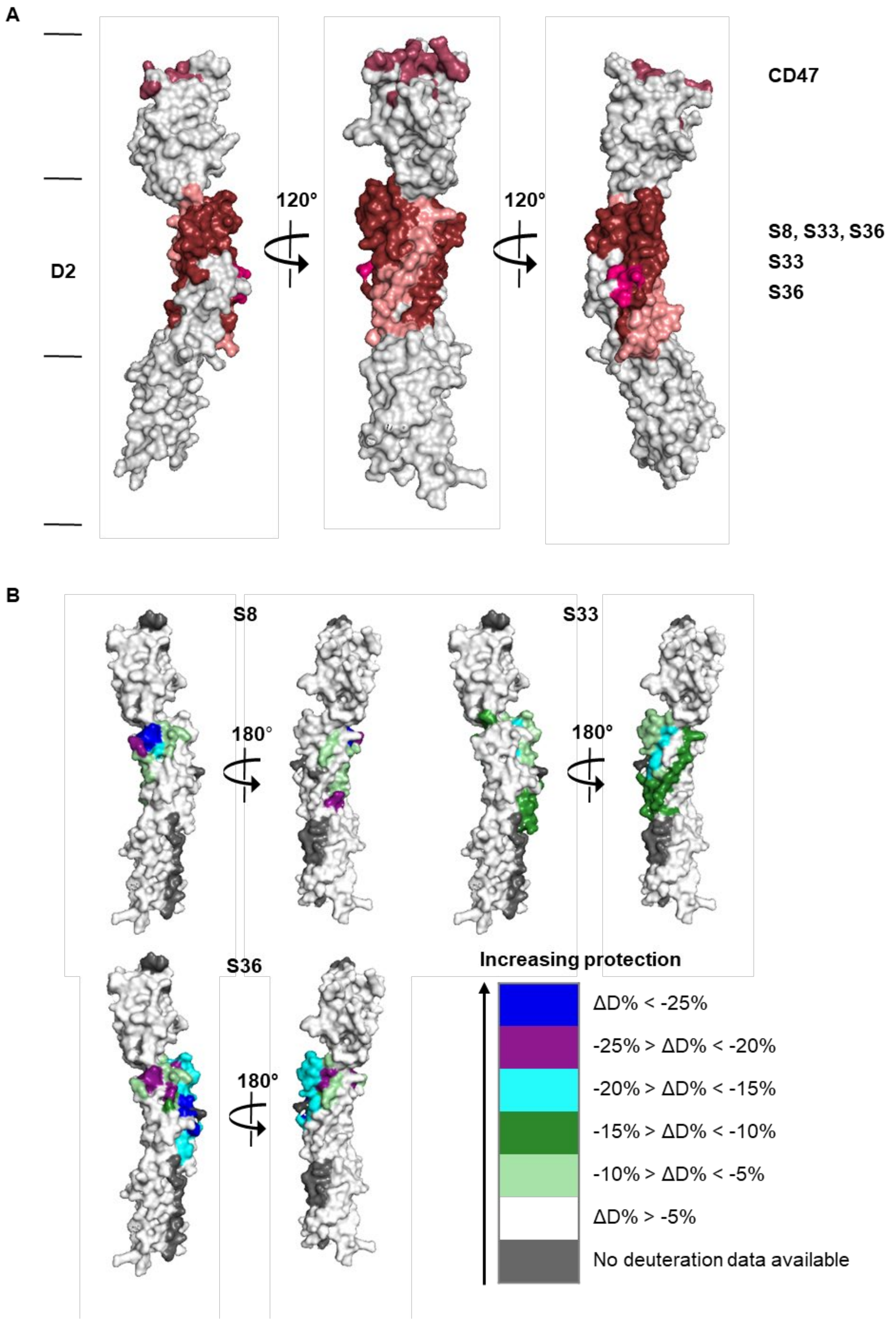

A
D2
120°
120°
CD47
S8, S33, S36
S33
S36
B
S8
S33
S36
180°
180°
180°
Increasing protection
ΔD% < -25%
-25% > ΔD% < -20%
-20% > ΔD% < -15%
-15% > ΔD% < -10%
-10% > ΔD% < -5%
ΔD% > -5%
No deuteration data available

Supplementary Figure 4. Detailed epitope mapping analysis of hSIRPαD2 Nbs by HDX-MS.

Localization of hSIRPαD2 Nbs binding epitopes by hydrogen-deuterium exchange mass spectrometry (HDX-MS).

A Surface structure model of hSIRPα (PDB 2wng) showing overlayed epitope mapping results of Nbs S8, S33 and S36.

B Surface structure model of hSIRPα (PDB 2wng) showing individual results of epitopes protected upon binding of hSIRPαD2 Nbs S8, S33 and S36 and different colors indicate the strength of protection (%ΔD).

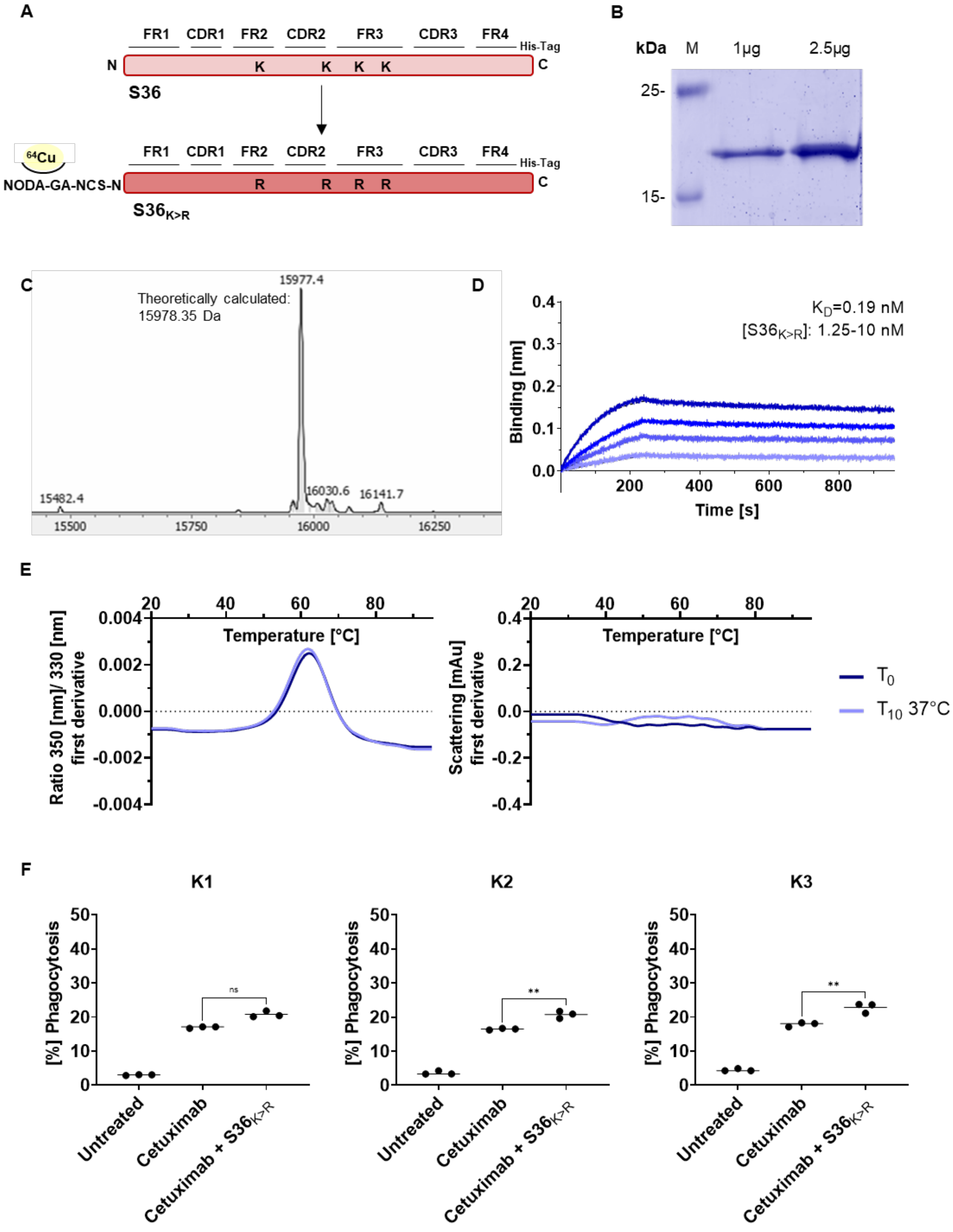

Supplementary Figure 5. Detailed characterization of the sequence optimized hSIRPα-S36$_{K>R}$ Nb for site-specific chelator conjugation.

A Graphical illustration of sequence optimization of hSIRPα-S36 Nb (S36$_{K>R}$) by changing lysine (K) to arginine (R) residues for site-specific chelator (p-NCS-benzyl-NODA-GA) conjugation.

B Expression and purification of hSIRPα-S36$_{K>R}$ Nb using immobilized metal affinity chromatography (IMAC) and size exclusion chromatography (SEC). Coomassie staining of 1 µg and 2.5 µg of purified and chelator-conjugated hSIRPα-S36$_{K>R}$ Nb is shown.

C Confirmation of identity and integrity by mass spectrometric (MS) analysis of chelator conjugated hSIRPα-S36$_{K>R}$ Nb (theoretically calculated molecular weight of 15978.35 Da).

D BLI-based affinity measurements of chelator conjugated hSIRPα-S36$_{K>R}$ Nb. Biotinylated hSIRPα was immobilized on streptavidin biosensors. Kinetic measurements were performed by using four concentrations of purified Nbs ranging from 1.25 nM to 10 nM.

E Stability analysis of chelator conjugated hSIRPα-S36$_{K>R}$ Nb by nanoDSF as fluorescence ratios (350 nm/330 nm) and light intensity loss due to scattering illustrated as first derivative before (T_0) and after 10 days of accelerated aging at 37°C (T_{10}). Data are shown as mean value of three technical replicates.

F Phagocytosis of DLD-1 cells by human monocyte-derived macrophages treated with anti-EGFR cetuximab and chelator conjugated hSIRPα-S36$_{K>R}$ Nb. Analysis of phagocytosis of hSIRPα-S36$_{K>R}$ Nb in combination with cetuximab of three different donors (K1, K2, K3). Data are shown as individual and mean value of three technical replicates. $p < 0.05$ was considered statistically significant (*) and marked as ** for $p < 0.01$, *** for $p < 0.001$, **** for $p < 0.0001$, non-significant results were marked with ns.

References

1. Traenkle B, Kaiser PD, Pezzana S, Richardson J, Gramlich M, Wagner TR, et al. Single-Domain Antibodies for Targeting, Detection, and In Vivo Imaging of Human CD4+ Cells. Frontiers in Immunology. 2021;12.

2. Virant D, Traenkle B, Maier J, Kaiser PD, Bodenhöfer M, Schmees C, et al. A peptide tag-specific nanobody enables high-quality labeling for dSTORM imaging. Nature Communications. 2018;9(1):930.

3. Ng PK, Li J, Jeong KJ, Shao S, Chen H, Tsang YH, et al. Systematic Functional Annotation of Somatic Mutations in Cancer. Cancer Cell. 2018;33(3):450-62.e10.

4. Maier J, Traenkle B, Rothbauer U. Real-time analysis of epithelial-mesenchymal transition using fluorescent single-domain antibodies. Scientific reports. 2015;5(1):1-13.

5. Traenkle B, Emele F, Anton R, Poetz O, Haeussler RS, Maier J, et al. Monitoring Interactions and Dynamics of Endogenous Beta-catenin With Intracellular Nanobodies in Living Cells*[S]. Molecular & Cellular Proteomics. 2015;14(3):707-23.

6. Arbabi Ghahroudi M, Desmyter A, Wyns L, Hamers R, Muyldermans S. Selection and identification of single domain antibody fragments from camel heavy-chain antibodies. FEBS letters. 1997;414(3):521-6.

7. Gramlich M, Maier S, Kaiser PD, Traenkle B, Wagner TR, Voglmeir J, et al. A Novel PNGase Rc for Improved Protein N-Deglycosylation in Bioanalytics and Hydrogen–Deuterium Exchange Coupled With Mass Spectrometry Epitope Mapping under Challenging Conditions. Analytical Chemistry. 2022;94(27):9863-71.

8. Wagner TR, Ostertag E, Kaiser PD, Gramlich M, Ruetalo N, Junker D, et al. NeutrobodyPlex—monitoring SARS-CoV-2 neutralizing immune responses using nanobodies. EMBO reports. 2021;22(5):e52325.

9. Masson GR, Burke JE, Ahn NG, Anand GS, Borchers C, Brier S, et al. Recommendations for performing, interpreting and reporting hydrogen deuterium exchange mass spectrometry (HDX-MS) experiments. Nature Methods. 2019;16(7):595-602.
